# Supplementary material for: Transient light-activated gene expression in Chinese hamster ovary cells
Source: BMC Biotechnol. 2021 Feb 4;21:13. doi: 10.1186/s12896-021-00670-1 (PMC7863527; doi:10.1186/s12896-021-00670-1)
Supplement: Supplementary file 1 — Additional file 1: Supplementary Figure S1. Percent eGFP-positive cells and MFI plotted against gate threshold. Supplementary Figure S2. qRT-PCR primer efficiencies. Supplementary Figure S3. Transient and lentiviral expression constructs. [file 12896_2021_670_MOESM1_ESM.pdf]

Transient light-activated gene expression in Chinese hamster ovary cells

Shiaki A. Minami[a] and Priya S. Shah[a,b,*]
[a] Department of Chemical Engineering
[b] Department of Microbiology and Molecular Genetics
University of California, Davis
* Correspondence: prsshah@ucdavis.edu, @shah_ps

Supplementary Information

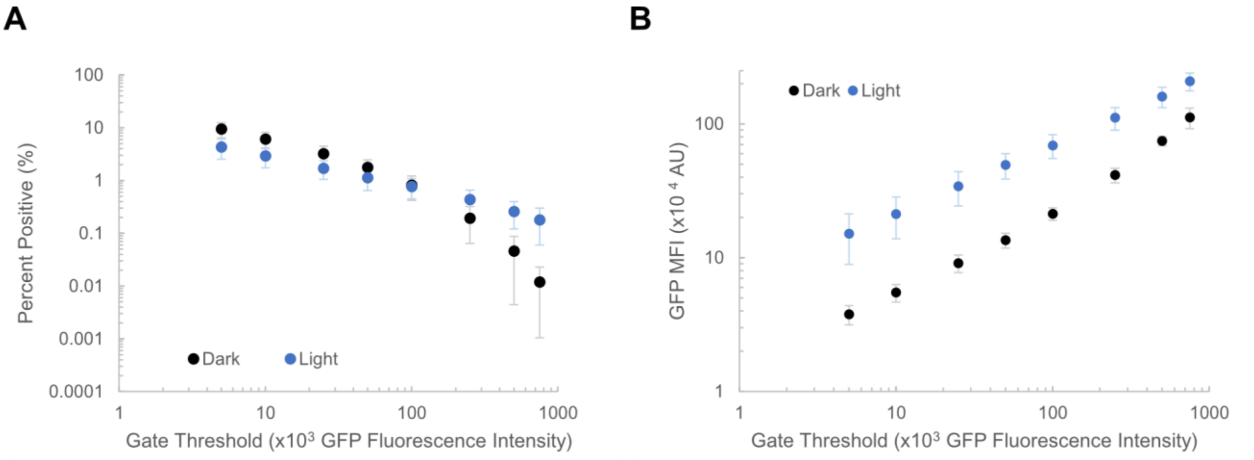

Figure S1. Percent positive cells and MFI plotted against gate threshold. (A) Number of cells crossing the eGFP-positive gate with increasing gate threshold. At high gate thresholds, the number of eGFP-positive cells are higher for the activated LACE system. At lower gate thresholds, the number of eGFP-positive cells are higher for the nonactivated LACE system. **(B)** GFP MFI of LACE cells plotted against gate thresholds. eGFP-positive cells are higher for the LACE system in the light for all gate thresholds.

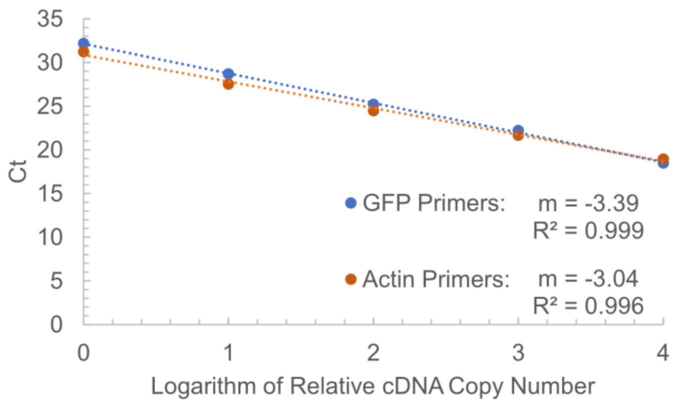

Figure S2. qRT-PCR primer efficiencies. Primer efficiencies for eGFP and Actin. eGFP and Actin primer efficiencies calculated from the slopes (m) of the log(cDNA) plots are 97.2% and 113%, respectively. Data are averaged from duplicate dilutions.

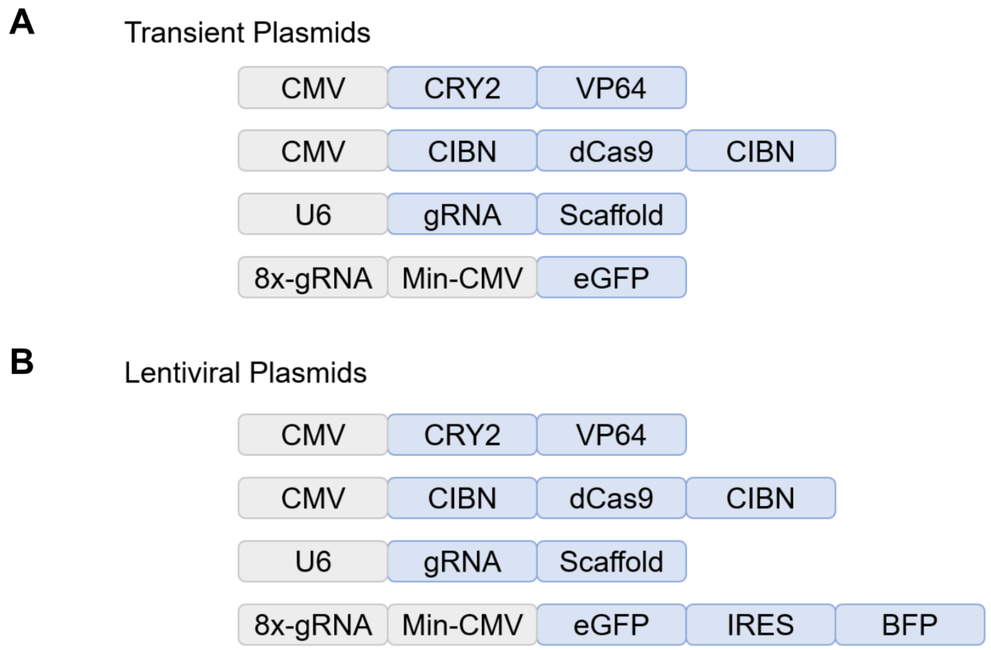

Figure S3. Transient and lentiviral expression constructs. (A) Transient plasmid expression constructs. CRY2 and CIBN are expressed by CMV promoters, gRNA is expressed by a U6 promoter, and GFP is expressed by a minimal CMV promoter, whose expression is activated by VP64. **(B)** Lentiviral expression constructs. All constructs except the minimal CMV construct are identical to the transient plasmids (**A**), except in a lentiviral backbone. The minimal CMV construct was modified to contain an additional IRES sequence and BFP to identify light-activated cells once the GFP is removed.
